# Women’s Preferences for Body Image Programming: A Qualitative Study to Inform Future Programs Targeting Women Diagnosed With Breast Cancer

**DOI:** 10.3389/fpsyg.2021.720178

**Published:** 2021-10-13

**Authors:** Jennifer Brunet, Jenson Price, Cheryl Harris

**Affiliations:** ^1^School of Human Kinetics, University of Ottawa, Ottawa, ON, Canada; ^2^Cancer Therapeutics Program, Ottawa Hospital Research Institute, Ottawa, ON, Canada; ^3^Institut du Savoir Montfort, Hôpital Montfort, Ottawa, ON, Canada; ^4^School of Psychology, University of Ottawa, Ottawa, ON, Canada

**Keywords:** breast cancer, survivorship, body image, qualitative, content analysis

## Abstract

**Purpose:** This paper describes women’s opinions of the attributes of the ideal body image program to inform the design, development, and implementation of future programs for those diagnosed with breast cancer.

**Methods:** Deductive-inductive content analysis of semi-structured interviews with 26 women diagnosed with breast cancer (mean age = 55.96 years; mean time since diagnosis = 2.79 years) was performed.

**Findings:** Participants’ opinions regarding the ideal body image program are summarized into five themes, mapping the *where* (community-based, hospital-based, or online), *when* (across the cancer continuum or at specific points), *how* (peer-led programs, professional help, events, presentations/workshops, resources, support groups), *what* (self-care, counseling and education for one self, education for others, support for addressing sexuality/sexual health concerns, and concealing treatment-related changes), and *who* (team approach or delivered by women, health professionals, make-up artists).

**Conclusion:** This study provides useful data on what women believe are the attributes of the ideal body image program, which can contribute to efforts aimed at developing and delivering body image programs for women diagnosed with breast cancer that prioritize their needs and preferences.

## Introduction

Worldwide, there are more than 2.1 million women diagnosed with breast cancer each year, making it the most common type of cancer diagnosed in women (Sung et al., 2021). Conventional treatments for breast cancer include surgery, chemotherapy, radiation, and hormone therapy (Senkus et al., 2015). Unfortunately, complete removal or changes to the size/shape/symmetry/sensation of one/both breast(s), hair loss, skin or fingernail discoloration, scaring, hot flashes, vaginal dryness, and weight gain/fluctuations are common side effects of these treatments (Ewertz and Jensen, 2011; Brunet et al., 2013). Such physical changes can have an enduring negative impact on women’s body image during and after treatment for breast cancer (Brunet et al., 2013). Approximately 17–33% of women recently diagnosed with breast cancer (Fobair et al., 2006) and 15–30% of women who have completed treatment for breast cancer experience some degree of body image concern (Anagnostopoulos and Myrgianni, 2009; Falk Dahl et al., 2010; Lyngholm et al., 2013), causing significant distress, impairing quality of life, and damaging sexual functioning (Fobair et al., 2006; Falk Dahl et al., 2010; Moreira and Canavarro, 2010; Lam et al., 2012). Consequently, body image has been deemed a “critical psychosocial issue for patients with cancer” (Esplen et al., 2021) and there has been a surge in research focused on developing programs for women to improve their body image and psychosocial adjustment during the course of the illness (Lewis-Smith et al., 2018b; Esplen and Trachtenberg, 2020; Esplen et al., 2020; Brunet and Price, 2021).

Two recent systematic reviews of studies with women diagnosed with breast cancer showed that body image programs comprised of psychotherapy, psychoeducation, or physical activity can have positive effects on body image (*d*s = 0.15–1.43, Lewis-Smith et al., 2018a; *g* = 0.50, 95% CI [0.08; 0.93], Sebri et al., 2021). Generally, programs reviewed with positive findings were delivered at different times during the course of the illness and adopted a multi-session, face-to-face, group format, though some online interventions have been shown to be effective in addressing body image distress among women diagnosed with breast cancer (Esplen and Trachtenberg, 2020). Although the studies reviewed are promising and support the importance of body image programs, there is insufficient applied evidence available to make recommendations at present for the type of body image program women diagnosed with breast cancer prefer. This is, in part, because there is a significant gap in the literature regarding the opinions of women toward body image programs.

Typically, body image programs are developed based on researchers’ opinions and available resources (e.g., time, financial), resulting in a variety of modes, formats, and content, and potentially programs that are not likely to match women’s needs and preferences (Arch et al., 2018). As such, it may be a challenging task for authorities and health professionals to discern what, how, and when to offer body image programs. Moreover, researchers have not consistently summarized the key methods and processes used to design body image programs based on women’s preferences (e.g., Hamzehgardeshi et al., 2017). Rather, the focus has often been on describing the mode, format, and content of programs to allow for replicability (e.g., Pinto et al., 2005). Thus, it remains unclear whether (and to what extent) researchers have involved women diagnosed with breast cancer in the creation of their programs. However, the potential of body image programs is likely to only be fully exploited when using an approach that takes women’s opinions and preferences regarding the mode and content, as well as the delivery format (e.g., face-to-face vs. online, individual vs. group-based, single- vs. multi-session) into account to enhance relevance, accessibility, and quality of implementation. Otherwise, a mismatch between what is designed and what women want can lead to poor usability, which can directly affect program efficacy. This issue can be resolved by conducting formative research to understand women’s opinions of the attributes of the ideal body image program.

Public involvement in the development, design, and delivery of programs is highly promoted (Mead and Bower, 2000; Kitson et al., 2013) and is vital to guide successful translation of the promising research findings of the benefits of body image programs into practice. Indeed, there is growing recognition of the need for researchers to adopt a user- or patient-centered design approach. A patient-centered approach to care typically involves identifying intended end-users of a program (e.g., women diagnosed with breast cancer) and then ascertaining and prioritizing their needs and requirements (Mead and Bower, 2000; Kitson et al., 2013). This can be done by consulting with end-users and involving them at specific points during the design process. To this end, researchers could employ qualitative methods to determine what women diagnosed with breast cancer seek from a body image program and explore their perceptions of the *what*, *when*, *where*, *who*, and *how* of the ideal body image program. The aim of the present qualitative study was therefore to explore women’s opinions of the attributes of the ideal body image program. This manuscript describes their opinions and presents implications for future program design, development, and delivery.

## Materials and Methods

### Participants and Procedures

This study used a qualitative interview design and adopted a constructivist paradigm approach wherein knowledge is considered introspective and co-developed based on individuals’ unique perspectives (Crotty, 1998). Ethics approval was obtained from the authors’ institutions, and the data reported in this paper were collected within a larger grounded theory study that aimed to explore the meaning of body image for women diagnosed with breast cancer and how they see their breast cancer experience as influencing their body image to develop a grounded theory of body image for this population (Brunet et al., 2021b). Adult women (18 years or older) who were fluent in English and had undergone treatment for breast cancer within the past 5 years were eligible to participate. No stipulations were made in terms of age limit or treatment type to ensure the inclusion of women with different opinions. Women were recruited from The Ottawa Hospital (Ontario, Canada) and through various Canadian-based cancer charities and organizations via their online forums, newsletters, bulletins, websites, and social media. Additionally, women were recruited through social networking sites and word of mouth. Women were provided with a monetary incentive of $25 CAD upon completion of the interview.

Twenty-seven women provided consent prior to completing the interview; one was removed from this study because she did not answer questions pertaining to the current aim due to time constraints. Data from the remaining 26 women (mean age = 55.96 ± 16.21 years; range = 25–81) were analyzed; Table 1 displays their characteristics and treatment details. All participants had their names replaced with an ID number (e.g., P1) to maintain confidentiality.

**TABLE 1:** Participants’ characteristics and treatment details (*n* = 26).

Variables	Descriptives
Age (years), M ± *SD*; range^a^	55.96 ± 16.21; 25–81
Married, *n* (%)^a^	18 (69.2)
White, *n* (%)^a^	22 (84.6)
Completed university/college/graduate school, *n* (%)^a^	20 (77)
Annual household income > $100,000 CAD, *n* (%)^a^	15 (53.8)
Body mass index (kg/m^2^), M ± *SD*; range^b^	28.08 ± 7.57; 18.95–57.24
**Cancer stage, *n* (%)^a^**	
0	1 (3.8)
I–III	20 (80.8)
IV	1 (3.8)
Do not remember	3 (11.5)
**Time since diagnosis (years), M ± *SD*; range^a^**	2.79 ± 1.64; 0.42–5.83
**Treatments received, *n* (%)**	
Surgery^a^	25 (96.2)
Chemotherapy^a^	15 (57.7)
Radiotherapy^a^	16 (61.5)
Hormonal^a^	16 (61.5)
Other^c^	8 (30.8)
**Comorbidities**	
Diabetes^a^	3 (11.5)
High blood pressure^a^	11 (42.3)
High cholesterol^a^	4 (15.4)
Arthritis^a^	8 (30.8)
Lung disease^a^	2 (7.7)
Osteoporosis^a^	6 (23.1)
Hip/joint replacement^a^	3 (11.5)
**Perceived physical health, *n* (%)^c^**	
Poor to fair	4 (15.3)
Good to very good	17 (65.4)
Excellent	3 (11.5)
**Perceived mental health, *n* (%)^a^**	
Fair	1 (3.8)
Good to very good	16 (61.6)
Excellent	8 (30.8)

*^*a*^*n* = 25; ^*b*^*n* = 23; ^*c*^*n* = 24.*

### Data Collection

Semi-structured interviews were conducted by a female research assistant in her 20’s with a Master’s degree, either in-person (*n* = 10), by telephone (*n* = 11), or via videoconferencing (*n* = 5). The latter two means were done to reduce barriers for participation (e.g., constrictive schedules) and permit interviews with women from a broader geographic area (Sedgwick and Spiers, 2009; Janghorban et al., 2014). An interview guide was created to facilitate discussion during the interviews regarding the research aim, while affording participants flexibility to elaborate on their opinions based on personal experiences (Denzin and Lincoln, 2011). A pilot interview was conducted with one woman diagnosed with breast cancer, who had expressed interest in the study after seeing it advertised on social media and met eligibility criteria, to trial and refine the interview guide. The wording of some questions was edited as a result to reduce the length of some of the questions, make questions as simple as possible, and remove jargon. Core questions that covered women’s opinions on what an ideal body image program would look like were: “*How do you envision the ideal body image workshop/intervention/program for women diagnosed with breast cancer?*” and “*What should be the aims, topics covered, settings/context, format, etc.?*” Based on women’s responses, the interviewer posed follow-up questions, including “*What should be the topics covered in a body image workshop/intervention/program?*”, “*What should be the format of a body image workshop/intervention/program?*”, “*What should be the setting/context of a body image workshop/intervention/program?*”, “*Who should deliver a body image workshop/intervention/program?*”, and “*When would be the ideal time to deliver a body image workshop/intervention/program?*”. She also probed for depth and clarity. Of note, the interviewer did not to provide participants with a definition of body image during the interview; rather, she was to encourage participants to interpret the meaning of body image for themselves and answer questions accordingly. Interviews were audio recorded and transcribed verbatim.

### Data Analysis

Transcripts of participants’ interviews were uploaded to NVivo and analyzed using deductive-inductive latent qualitative content analysis (Graneheim and Lundman, 2004) by two independent researchers. Both researchers (1) read each interview to acquire an overall understanding of content related to the research aim; (2) read each interview again while noting repeated viewpoints that could represent meaning units; (3) created and discussed a coding scheme to support subsequent coding of meaning units; (4) coded each interview based on these meaning units; and (5) extracted condensed meaning units out of meaning units with regard to the context. Those condensed meaning units that were related in terms of their meaning and content were then merged and abstracted into themes.

### Reflexivity

The research team (i.e., authors, research coordinator, and the interviewer) reflected on their positions throughout the research process. They acknowledge that none had been diagnosed with breast cancer at the time of this study. As such, they were keenly aware of the differences between themselves and the interviewed women, especially in terms of disease history and age. Nonetheless, they had empathy for the interviewed women and the challenges they had faced as a result of having been diagnosed with breast cancer. Moreover, the interviewer felt compelled at times to reassure women and struggled to curb her urge to offer support in a matter that would take her beyond her role. To avoid this and protect against biases, she spent time debriefing with the first author after interviews.

## Results

Participants clearly had an overwhelming desire to access a body image program. Yet, the data collected in this study suggest they had a range of opinions regarding the attributes of the ideal body image program. Participants’ opinions were summarized into five themes reflecting their preferences for the *where*, *when*, *how*, *what*, and *who* of the ideal body image program. Themes are summarized in Table 2 (with a selection of representative quotes from participants) and outlined below.

**TABLE 2:** Summary of attributes for the ideal body image program.

Attributes	Program specifics	Quotations	Divergences
*Where* we want to attend a program	• Community-based (*n* = 2) • Online (*n* = 4) • Hospital-based (*n* = 3)	*People don’t really want to have to come to the city. They want to be able to do it in their community. [*…*] You don’t want to have to travel and I think that’s a big, big thing*. P2 *Everybody has such a busy life. It would be great to have some help online. With the younger women, more and more, everybody needs that. And between treatments and work and everything, you would like to have something at your fingertips*. P10 *Just a website that someone could say go a have a look at what you could hope for or what happened to other people and you could have an idea of what you’re facing*. P19 *It would make more sense if it happened at the hospital.* P18	Non-hospital (*n* = 5) *I’d have a room that’s not sterile because you’ve been in these sterile situations over and over and over again. Something that’s like warm and inviting and cozy. [*…*] Not in a hospital*. P16 *It costs $13 every time you go. [*…*] People have to then travel from wherever they live to come to the hospital and fight to find parking, and have to pay for parking, and it’s just wrong. As a patient, you pay so much for parking already, so people aren’t going to go*. P2
*When* we want a program	• Across the cancer continuum (*n* = 3) • Pre-diagnosis (*n* = 1) • At or immediately post-diagnosis (*n* = 4) • Post-surgery (*n* = 3) • During treatment (*n* = 3) • Post-treatment (*n* = 6)	*I think before treatment starts is a good one. [*…*] Then part way through, a check-up with women. [*…*] And then at the end of treatment.*P21 *If after surgery you’re in the hospital [*…*] just having somebody that’s engaging you on this topic*.P26 *While going through treatment.*P17 *Stopping treatment also is a huge up hill. It’s like “now what?” That would be a good time for the workshop*. P14	Not during treatment (*n* = 2) *Personally, as a breast cancer patient, if you organize a workshop on body image, I would laugh. It’s just not where I’m at. I’m not going willingly go to a workshop while I’m in treatment.* P26
*How* we want a program (i.e., format)	• Peer-led program (*n* = 7) • One-on-one counseling (*n* = 1) • Special events (*n* = 5), presentations/workshops (*n* = 10) • A compilation of resources women can access on their own (*n* = 2) • Support groups for women that facilitate discussion and encourage friendly relationships (*n* = 8)	*I feel like everybody diagnosed with cancer should have a one-on-one mentor with someone who went through it*. P18 *If the hospital were to put on something about breast cancer, like body image, I think women would come out of the woodwork to do that.* P23 *Maybe some lectures with subjects [where] I don’t have to open my mouth, I just listen and learn.* P17 *I wish there was a sort of formal class*. P9 *A workshop that essentially helps you to restore your physical self, your appearance, and helps to promote feeling good about yourself*. P1 *I’m a literature person*. P15 *I think support group would be a really important part. [*…*] Different support groups for different needs, for different women*. P13	
*What* we want in a program	• Self-care: – Promoting healthy eating (*n* = 3) – Encouraging physical activity for improved health and wellbeing (*n* = 6) – Giving sleep advice (*n* = 3) – Discussing body size, shape, and weight management (*n* = 2) • Counseling and education for oneself: – Promoting self-compassion (*n* = 12) – Focusing on inner strength more so than appearance (*n* = 5) – Building resistance to body-based stereotypes/prejudices (*n* = 4) – Supporting spiritual growth – which can exist within or independent of religion – to feel more connected with life (*n* = 3) – Building confidence (*n* = 1) – Discussing typical physical changes (*n* = 6) – Learning to deal with stress and mental health issues (*n* = 2) – Discussing sexuality/sexual health (*n* = 5)	*The male part of the equation really needs some help and understanding. Understanding how a female feels, what she’s going through, and what she might want as well*. P13 *I think eating properly, sleeping properly, keeping your body weight within a good range of what it should be*. P12 *Having a makeup artist, having beautiful clothes that you can wear [*…*]. Wearing that piece of clothing, that beautiful dress, that makes you feel good. [*…*] So doing that type of workshop, that self-care workshop*. P17 *I wish there was a sort of formal class or something that I could have gone to that was designed for strength, stretching, and movement. You still need that strength to move and to be engaged in your life. [*…*] I think that’s a critical piece of accepting your body*. P9 *I would try to tell them to be happy with themselves and that really how you look is not that important to other people*. P11 *If a workshop were to focus on strategies of accepting yourself and maybe strategies of dealing with messaging or pressures, whether that be, at large, sort of media and public images, or from friends or family. That could be helpful*. P3	Do not focus on make-up and clothing (*n* = 2) *Do you know the Look Good, Feel Better? I had a really hard time with that. I didn’t enjoy it and part of me gets it, but another part of me doesn’t. I guess it’s because I’ve never been a big make-up person and all that kind of stuff. So it wouldn’t be like that is what I’m saying*. P14
	• Education for others (*n* = 3) • Support for addressing sexuality/sexual health concerns (*n* = 3) • Concealing treatment-related changes (*n* = 4)	*Downgrading those stereotypes and letting the participants know that they have control over how they see their body and what they can do and that they don’t have to fit in that mold that’s been set by society*. P5 *I guess just allow people to know that “Here’s 10 ladies who had lumpectomies and here’s what their breasts look like afterward” so when you get your lumpectomy and you look and see what you’ve got you can think well that’s about average. If you look at it without knowing what other lumpectomies look like, you might think “Oh shit that looks like crap.” But when you can compare it to other women, you might get a sense of well that’s as good as they can do I guess*. P19 *A workshop that focuses on women really working on self-acceptance and not judging. Finding their own strengths and beauty, their own natural beauty, and defining it in different terms*. P13 *Focus on inner strength. How do we make a person feel to how do we make a person to accept these difficult circumstances and how to make that person feel that no you have not lost anything*. P27 *I would definitely start with a mental health component, that would be number one for me*. P14 *Maybe you have somebody who is a body image specialist who helps improve sexual awareness for instance, because that’s a very big topic around women my age who have breast cancer, but where do we find those resources? Between ourselves, we get our own speakers, we get people to talk to us, we go for talks and stuff like that, but having somebody organize something like that for us, so if there’s a professional that you can also ask those tough questions to would be helpful*. P26 *My main issue is weight so if I want to optimize my body image, I do have to work toward losing a little bit of weight and firming up the arms, the legs, and the abdomen area*. P5	
*Who* we think should deliver a program	Team approach – women diagnosed with breast cancer and health professionals (*n* = 4) Women diagnosed with breast cancer (*n* = 16) Health professionals (*n* = 11) Make-up artists and/or clothing stylists (*n* = 1)	*I think your best bet is a mix of medical professionals and peers; people who have been through it themselves. I would say both*. P14 *For me, it would be helpful if I heard it from people who have gone through it. It touches me more from people who have experienced what I have experienced and who have overcome their insecurities and have back their self-confidence because that’s what you strive to be like.* P25 *A nurse or a doctor could talk about health or the issues about the body image*. P5	
		*I think a nurse. [*…*] They’re really helpful, very knowledge, and really a little bit more in tune with the patients and their emotional needs*. P16 *Have somebody who is a body image specialist*. P26 *Having a professional makeup artist there. [*…*] Someone from a prosthetics or a mastectomy store who’s worked with women in fitting bras and undergarments and bathing suits and gowns and stuff like that*. P7	Non-health professionals (*n* = 5) *Not a healthcare professional because healthcare professionals are not, in my opinion, exposed to these things*. P27 *Surgeons are technical. So I would say no*. P7

### *Where* We Want to Attend a Program

This theme refers to participants’ preferred location for a body image program. Based on participants’ collective accounts, body image programs should be delivered across different locations because “*there’s never going to be a convenient place for everyone at different times*.” Of the 26 participants, two expressed interest in a body image program that could be undertaken within the community (i.e., public spaces close to home) for convenience. Four expressed interest in a body image program that could be delivered online to obtain easily accessible information at convenient times at their preferred location. Three mentioned that a body image program could be delivered in-hospital because it “*makes more sense*” and because they had “*become accustomed to medical settings*.” However, they did not necessarily feel it was the most appropriate setting. Moreover, five participants voiced concerns about having to go to the hospital for a body image program because they thought of the hospital as “*such a sterile place that nobody wants to go to*” and added that parking would likely deter participation.

### *When* We Want a Program

This theme describes participants’ preferences for the timing of a body image program. Participants had a variety of opinions, highlighting that differential timing may be needed for different women. Only three participants were interested in accessing a body image program across the cancer continuum, suggesting there should be “*several sections. There would be a newly diagnosed section, then there’d be the treatment time, then there’d be the post-treatment time, and then there’d be the rest of your life*.” Only one participant was of the opinion that a program offered pre-diagnosis would be of interest because “*if you’re coming from a place of negative body image and then you have breast cancer, I mean, you would need a lot of help*.” However, similar to two others, she also believed post-surgery would be good timing. Others described a preference for a program at diagnosis or immediately after (*n* = 4), during treatment (*n* = 3), or post-treatment (*n* = 6). Further supporting post-treatment timing, two participants expressed a negative opinion about having a body image program during treatment because they believed women would have other priorities (e.g., completing treatment). Thus, overall, participants were of the opinion that women should be able to undertake a body image program when they are comfortable doing so.

### *How* We Want a Program

This theme refers to the format participants were interested in for a body image program. Ten participants were interested in attending presentations or workshops focused on body image, and an additional five suggested special events. Whilst different terms were used by participants, they described presentations, workshops, and special events as one-time occurrences wherein information pertaining to body image (e.g., self-care, strategies for self-acceptance) could be shared with attendees to facilitate knowledge. Yet, it is unclear what the size of the audience should be as “*there’s safety in small groups but sometimes there’s anonymity in big groups. So you might be doing the same things, but have two different kinds of workshops and you give them the option of what ones they would rather [attend]*.” Another option mentioned was to have a peer-led program (*n* = 7) or support group for women that facilitates discussion and encourages friendly relationships (*n* = 8). This was driven by participants’ belief that camaraderie would be built with other women diagnosed with breast cancer and that peer social support would be facilitated. Others, however, were more interested in receiving individual guidance or support such as through one-on-one counseling (*n* = 1) or by receiving a compilation of resources women can access on their own (*n* = 2). Whilst divergent opinions were shared by participants, they generally agreed that, regardless of the format, a one-size fits all approach would not work as “*you’d probably have to cover quite a wide range because everybody has different problems depending on your age, your situation, and things are different*.”

### *What* We Want in a Program

This theme describes what topics participants would like to see covered in a body image program. Participants identified 13 topics, none of which were unanimously recommended, highlighting the personal nature of body image and the potential need for tailoring. Despite the diversity in participants’ preferred topics, these could be organized into five areas: self-care, counseling and education for oneself, education for others, support for addressing sexuality/sexual health concerns, and concealing treatment-related changes. Of the 26 participants, 14 mentioned an interest in self-care, including promoting healthy eating (*n* = 3), encouraging physical activity for improved health and wellbeing (*n* = 6), giving sleep advice (*n* = 3), and discussing body size, shape, and weight management (*n* = 2). Of note, many of these participants expressed interest in multiple aspects of self-care. Within the realm of counseling and education, two key topics prevailed. Twenty-five participants expressed a desire for a program aimed at accepting and embracing their new normal, either by promoting self-compassion (*n* = 12), focusing on inner strength more so than appearance (*n* = 5), building resistance to body-based stereotypes/prejudices (*n* = 4), supporting spiritual growth – which can exist within or independent of religion – to feel more connected with life (*n* = 3), and building confidence (*n* = 1). Another 13 mentioned counseling and education related to addressing the side-effects of cancer treatment, including discussing typical physical changes (*n* = 6), learning to deal with stress and mental health issues (*n* = 2), and discussing sexuality/sexual health (*n* = 5). Raising awareness of how body image affects women (*n* = 3), especially with partners, was also mentioned and thus necessary to “*focus on the people around, the support system, and yes, educate men. [*…*] Like have him attend a meeting, educate him, this is how your wife is feeling*.” In addition to discussing sexuality/sexual health, three participants mentioned wanting help addressing sexuality/sexual health concerns to help improve their body image. Finally, four participants expressed interest in receiving make-up and clothing tips for coping with body image changes, though two others held negative opinions toward make-up and clothing-oriented topics.

### *Who* We Think Should Deliver a Program

This theme refers to the person(s) who participants felt should deliver a body image program to women diagnosed with breast cancer. Because the person(s) delivering the program is central to what and how the content is delivered, most participants (*n* = 16) preferred that women previously treated for breast cancer be involved in delivering the program because “*you need somebody that’s been in the trenches*.” Similarly, four said that a promising option could be to have women previously treated for breast cancer partner with a health professional to deliver the program. The rationale provided was that “*some people really like having the authority of having a medical degree behind the person speaking. Some people really like that and have difficulty if they don’t have that. Other people prefer a more peer type interaction with the person presenting. So those are dichotomies. How are you going to do that unless you have two people speaking, which obviously might work*.” Generally, participants who desired that women diagnosed with breast cancer be involved in delivering the program were driven by the belief that having a shared experience was an essential component of a body image program. Eleven participants expressed an interest in having health professionals such as doctors (*n* = 6), nurses (*n* = 5), dieticians/nutritionists (*n* = 2), psychologists (*n* = 3), social workers (*n* = 4), fitness professional or physiotherapists (*n* = 3), or sex therapists (*n* = 2) deliver the program. Only one participant mentioned having a make-up artist and/or clothing stylist deliver the program. Given this diversity, delivering a body image program may require a multi-disciplinary team approach, whereby women previously treated for breast cancer partner with health professionals to design and deliver programs to meet an assortment of needs and preferences.

## Discussion

Previous research indicates that it is not uncommon for differences to exist between end-users’ needs and preferences and existing programs/interventions (Short et al., 2014; Cadmus-Bertram et al., 2020). The aim of the present qualitative study was to gain insight into the attributes of the ideal body image program for women diagnosed with breast cancer from their perspective. Through a series of open-ended questions, participants shared their opinions on the *where*, *when*, *how*, *what*, and *who* of the ideal body image program, which has implications for future program design, development, and delivery. Most notably, participants’ accounts confirm that topics covered in previous body image programs are of relevance, though there are other topics that should be covered as well. Moreover, participants indicated that the ideal body image program would be offered across the cancer continuum so that women can attend when they are ready. Finally, participants suggested that alternatives for when, where, who, and how be considered rather than having a standard program. Indeed, they underscored the need for diverse programs in terms of content and delivery to cater to different audiences and contexts.

Within the “*What* we want in a program” theme, participants identified 13 topics they wished a body image program would cover, which could be grouped into self-care, counseling and education for oneself, education for others, support for addressing sexuality/sexual health concerns, and concealing treatment-related changes. It was encouraging to find that participants were interested in topics previously covered in existing body image programs comprised of psychotherapy, psychoeducation, or physical activity (Esplen et al., 2018; Esplen and Trachtenberg, 2020; Lewis-Smith et al., 2018b). It is, perhaps, not surprising because psychotherapy and psychoeducation have been shown to help women challenge negative thinking and self-talk, modify unhealthy thoughts, feelings, and behaviors (i.e., cognitive restructuring), receive empathetic and non-judgmental support, while also exploring and addressing factors that contribute to negative body image (Neff, 2003). Whereas physical activity, while often promoted as a strategy for weight management due to its effects on body weight and shape (Zhu et al., 2016), can help women appreciate their bodies’ strength and respect it as it is despite weight, shape, and perceived imperfections (Lewis-Smith et al., 2018b). Nevertheless, the relationship between physical activity and body image is tricky because women may withdraw if they do not see changes in their body composition and because appearance-based physical activity motivation (i.e., the extent to which physical activity is pursued to influence weight or shape) weakens the association between physical activity and positive body image (Homan and Tylka, 2014). Thus, emphasis within a body image program should be placed on building women’s awareness of their physical capabilities by helping them focus on what their body can do, learn new physical activities, appreciate what they can do with their body and enjoy being active, and accept that healthy bodies come in different shapes and sizes in order to reduce focus on physical appearance. As well, it may be helpful to emphasize non-appearance-based physical activity motivation (e.g., health, enjoyment) and encourage women to attend to the sensations that can result from physical activity (e.g., improved mood, Homan and Tylka, 2014). Still, one topic identified by participants seems to have received relatively little attention despite mounting evidence on its importance – sexuality/sexual health post-treatment (Male et al., 2016). The extant literature and the diversity of topics identified in this study underscores the personal nature of body image and the potential need for tailoring. Accordingly, greater improvements in body image may be seen if women are provided with a program covering a topic (or topics) that align with their needs and preferences such as those mentioned with in the “*What* we want in a program” theme (Kwan et al., 2010).

Surprisingly, little is known about the effectiveness of body image programs based on *who* delivers the program. In this study, within the “*Who* we think should deliver a program” theme, participants mostly suggested a multi-disciplinary team approach whereby women diagnosed with breast cancer and health professionals co-facilitate the program. In comparison, most published body image programs have been delivered by trained researchers or health professionals (Lewis-Smith et al., 2018b). This is not unexpected as women diagnosed with breast cancer have expressed interest in programs guided by experts such as fitness professionals or healthcare providers (Wong et al., 2018), and the current findings are partly consistent with this observation. However, it may be beneficial to involve more women diagnosed with breast cancer in the delivery of body image programs. A clear benefit of doing so is that these women have first-hand experience. This said, the instructor/facilitator is central to how the content of a program is delivered which further has the potential to influence how it is received by end-users. In fact, researchers have called attention to the interpersonal style of the facilitator as important to the receptiveness of participants to information presented in an intervention (Hagger and Hardcastle, 2014; Hardcastle et al., 2017). Moreover, participants in this study highlighted the importance of credibility and knowledge about the topic alongside the importance of developing rapport and being able to speak simply without relying on technical language or jargon to explain concepts. As such, when designing body image programs in the future, it is important to not just be mindful of *who* is delivering the program, but also their capacity to connect with and convey information to the audience. Indeed, if employing a multi-disciplinary team approach, it would be necessary to ensure all involved parties respect the individual expertise and experience that each instructor/facilitator brings to the program, are comfortable interacting with other instructors/facilitators and participants, and are prepared and not overburden by participation in the program. As a systematic review of the impact of patient and public involvement on those involved in health and social care illustrated (Brett et al., 2014), this can be a challenge, though it is typically worthwhile for participants as multiple instructors/facilitators provide more diverse perspectives and knowledge.

The physical environment and setting are also important components to consider when designing a body image program. Within the “*Where* we want to attend a program” theme, some participants suggested a community-based setting. The participants placed importance on creating a welcoming setting that was comfortable and convenient. Although the hospital was considered a suitable location to some because of familiarity with the location, the expensive parking and clinical environment reduced the appeal of the location. Indeed, as researchers have previously noted (Blaney et al., 2013; Browall et al., 2018; Clifford et al., 2018), appropriate locations for supportive care programs and services are those located in close proximity to potential participants’ homes to enhance program attendance because home, work, and family obligations, particularly for women (Mailey et al., 2014; Browall et al., 2018), may limit their ability to attend programs. To maintain a free or low-cost program, familiar community spaces (e.g., local churches, libraries, public schools, community centers) might be potentially suitable locations. However, not all participants desired for a body image program to be delivered in-person as the added travel time may make it difficult to attend. This concern is echoed in the extant literature (Brunet et al., 2021a), which has resulted in alternative modes of delivery for body image programs, such as online programs (Esplen and Trachtenberg, 2020). In line with previous research (Phillips et al., 2017, 2019), online was identified as a favorable mode of delivery among some participants. The use of technology as a mode of delivery could provide an opportunity to reach a larger target population (Prutzman et al., 2021) and may open the door for a more diverse crowd of participants and instructors/facilitators.

Regardless of the mode of delivery (e.g., in-person, online), participants expressed a desire for group discussions and interactive activities that facilitate peer engagement, print or digital materials (e.g., books, fact sheets, or pamphlets), and one-on-one counseling. In particular, a large proportion of participants expressed a desire to interact with other women diagnosed with breast cancer in order to have opportunities to ask questions and share experiences. Notably, interaction with others has been highlighted as an important component of creating an optimal experience in psychological and behavioral interventions for adults diagnosed with cancer (Cavallo et al., 2014; Lee and Suh, 2018; Lewis-Smith et al., 2018b). As research expands to examine the acceptability and effectiveness of digital delivery methods, such as telehealth, as means to offer accessible programs, it will be critical to maintain social interaction opportunities for participants.

### Limitations and Strengths

The limitations of this study should be acknowledged. It only included English-speaking women living in a high-income country. Relatedly, the high education and lack of racial and ethnic diversity in the sample is another limitation. It is possible that women living in lower income countries, women with lower education, or women from different racial or ethnic groups may have different opinions and preferences. Also, participants were post-treatment. By interviewing women post-treatment, it is possible that certain opinions were influenced by reduced recall. Prospective, longitudinal studies are warranted to understand women’s opinions at different points in time. Similarly, participants had completed treatment within the past 5 years. Accordingly, it will be necessary to determine if the opinions and preferences expressed by participants in this study are echoed by women further away from treatment completion. Additionally, data were analyzed to aid in the development, design, and delivery of future body image programs for women diagnosed with breast cancer that meet their needs and align with their interests and preferences. It remains to be seen if such programs are just as effective as others tested in the literature. However, the strengths of this study were its sample size, inclusion of women with a range of ages and cancer experiences to support a diversity of perspectives, and analysis conducted by two researchers experienced in qualitative methodology.

## Conclusion

The objective of this study was to investigate women’s perspectives of the ideal body image program for women diagnosed with breast cancer. Participants’ accounts suggest that a body image program may be an effective way to help them feel better about their bodies in a world that constantly makes them focus on their perceived flaws, personal inadequacies, and failures. Although one specific formula for how to design, develop, and deliver a body image program to women diagnosed with breast cancer may not exist, the findings of this study lay the foundation for the development, design, and delivery of a more diverse and tailored approach to such programs. Specifically, findings provide a starting point for *what* (self-care, counseling and education for oneself, education for others, support for addressing sexuality/sexual health concerns, and concealing treatment-related changes), *when* (across the cancer continuum or at specific points), *where* (community-based, hospital-based, or online), *who* (team approach or delivered by other women, health professionals, make-up artists or stylists), and *how* (peer-led programs, professional help, events, presentations/workshops, resources, support groups) that should be considered when developing, designing, and delivering body image programs targeting women diagnosed with breast cancer. It is hoped this information will help in the creation of more meaningful, relevant, useful, and usable programs for this growing segment of the population.

## Data Availability Statement

The datasets presented in this article are not readily available because the data cannot be shared as participants were assured that their data would be kept private and confidential to the extent permitted by law and that only the research team would have access to the data. Requests to access the datasets should be directed to JB, Jennifer.Brunet@uottawa.ca.

## Ethics Statement

This study involving human participants was reviewed and approved by the Ottawa Health Science Network Research and the University of Ottawa Ethics Boards. Participants provided their written informed consent to participate in this study.

## Author Contributions

JB conceptualized and designed the study, oversaw the acquisition of the data, contributed to the interpretation of the data, and drafted the manuscript. JP led the analysis and interpretation of the data, helped draft sections of the manuscript, and critically revised the manuscript. CH assisted with the conceptualization of the study, participant recruitment, and revised the manuscript. All authors gave final approval of the version to be published and agreed to be accountable for all aspects of the work.

## Conflict of Interest

The authors declare that the research was conducted in the absence of any commercial or financial relationships that could be construed as a potential conflict of interest.

## Publisher’s Note

All claims expressed in this article are solely those of the authors and do not necessarily represent those of their affiliated organizations, or those of the publisher, the editors and the reviewers. Any product that may be evaluated in this article, or claim that may be made by its manufacturer, is not guaranteed or endorsed by the publisher.
